# Identifying the Viral Genes Encoding Envelope Glycoproteins for Differentiation of *Cyprinid herpesvirus 3* Isolates

**DOI:** 10.3390/v5020568

**Published:** 2013-01-31

**Authors:** Jee Eun Han, Ji Hyung Kim, Tristan Renault, Casiano Choresca, Sang Phil Shin, Jin Woo Jun, Se Chang Park

**Affiliations:** 1 Laboratory of Aquatic Biomedicine, College of Veterinary Medicine and Research Institute for Veterinary Science, Seoul National University, Seoul, 151-742, Republic of Korea; E-Mails: yellowjeeeun@hanmail.net (J.E.H.); cassey2002@yahoo.com (C.C.); charisma-shin@hanmail.net (S.P.S.); advancewoo@hanmail.net (J.W.J.); 2 Korea Ocean Research and Development Institute, Ansan, 426-744, Republic of Korea; E-Mail: mephisto81@hanmail.net; 3 Institut Francais de Recherche pour l’Exploitation de la Mer (IFREMER), Laboratoire de Génétique et Pathologie (LGP), Avenue de Mus de Loup, 17390 La Tremblade, France; E-Mail: Tristan.Renault@ifremer.fr

**Keywords:** *Cyprinid herpes virus 3*, glycoprotein gene, Koi herpesvirus, microsatellite, polymorphism, phylogenetic tree

## Abstract

*Cyprinid herpes virus 3* (CyHV-3) diseases have been reported around the world and are associated with high mortalities of koi (*Cyprinus carpio*). Although little work has been conducted on the molecular analysis of this virus, glycoprotein genes identified in the present study seem to be valuable targets for genetic comparison of this virus. Three envelope glycoprotein genes (ORF25, 65 and 116) of the CyHV-3 isolates from the USA, Israel, Japan and Korea were compared, and interestingly, sequence insertions or deletions were observed in these target regions. In addition, polymorphisms were presented in microsatellite zones from two glycoprotein genes (ORF65 and 116). In phylogenetic tree analysis, the Korean isolate was remarkably distinguished from USA, Israel, Japan isolates. These findings may be suitable for many applications including isolates differentiation and phylogeny studies.

## 1. Introduction

The production of ornamental fish is an important component of the aquaculture industry in several countries [1]. In particular, the commercial production of koi (*Cyprinus carpio*) has grown to become a major part of the pet industry within the past few decades, and koi trade plays an important role in meeting the increasing global demand [2].

Koi herpesvirus (KHV) disease, a highly contagious and virulent disease of carp and koi, was first recognized and documented in Germany in 1997 [3]. KHV is a double-stranded DNA virus belonging to the order *Herpesvirales*, family *Alloherpesviridae*, under the formal designation *Cyprinid herpes virus 3* (CyHV-3) [4,5].

Members of the order Herpesvirales consist of a linear, double-stranded DNA genome packaged within an icosahedral capsid of characteristic architecture that is surrounded by a proteinaceous tegument layer and finally by a host-derived envelope containing virus glycoproteins [6]. Glycoproteins, which are major component of the virion envelope and relevant for virus-host cell interactions and for the host immune response [7,8,9], have been targets for serological markers or vaccine candidates and have also been used for a phylogenetic analysis in the order *Herpesvirales* [10,11,12].

In this study, three CyHV-3 envelope glycoprotein genes were amplified from Korean isolate and the sequences were compared with previously reported CyHV-3 from the USA, Israel and Japan. Subsequently, microsatellite polymorphisms were analyzed from two glycoprotein genes and phylogenetic trees based on these genes were described.

## 2. Results and Discussion

### 2.1. Analysis of CyHV-3 Envelope Glycoprotein Sequences

**Figure 1:**
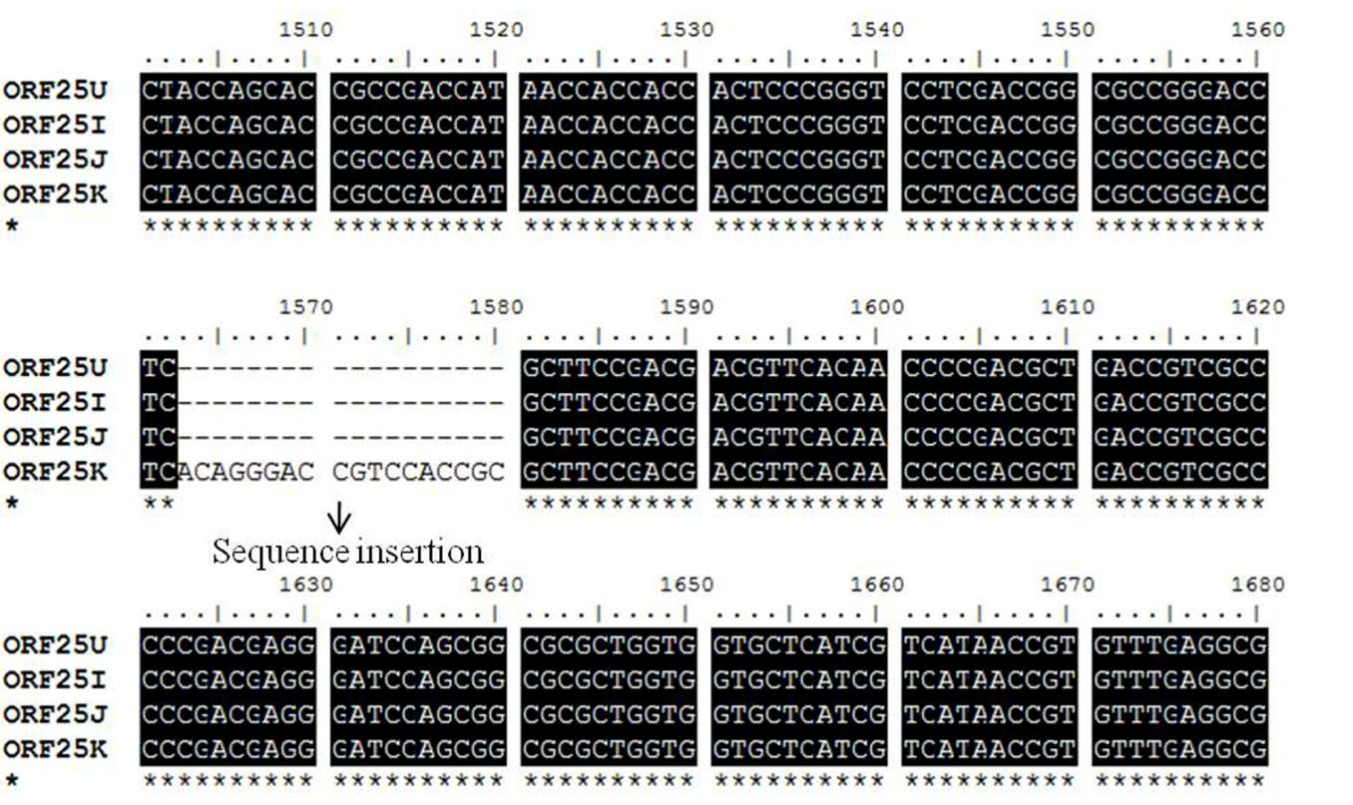
Nucleotide sequences of the glycoprotein gene (open reading frame [ORF]25) of the Korean CyHV-3 (KHV-K) and those of the USA (KHV-U), Israel (KHV-I), and Japan (KHV-J) CyHV-3 were aligned using BioEdit software. A sequence insertion (18 bp) was observed in Korean CyHV-3 (KHV-K).

**Figure 2:**
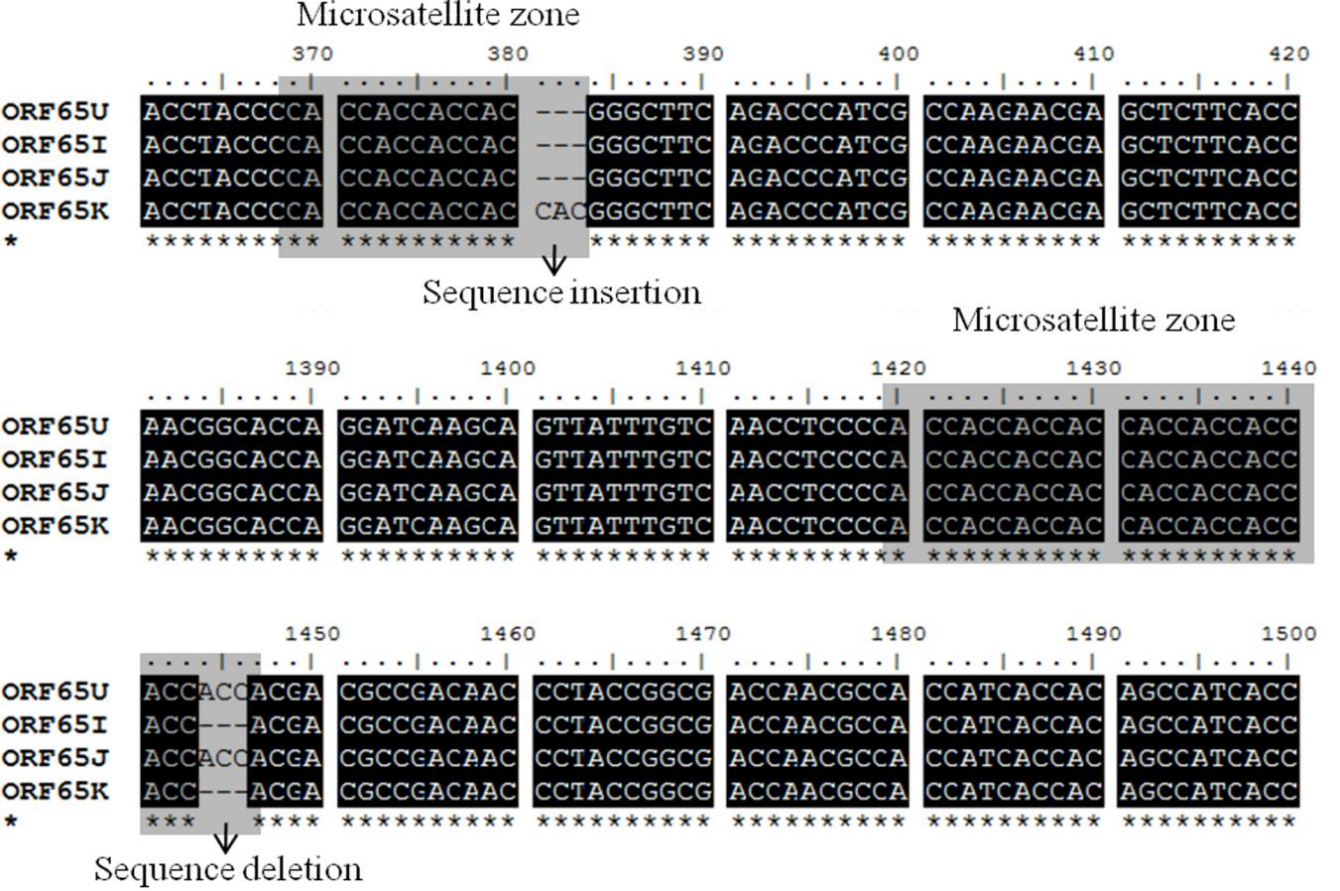
Nucleotide sequences of the glycoprotein gene (open reading frame [ORF]65) of Korean CyHV-3 (KHV-K) and those of the USA (KHV-U), Israel (KHV-I), and Japan (KHV-J) CyHV-3 were aligned using BioEdit software. Two microsatellite zones were observed, and both sequence insertions and deletions were observed in KHV-K.

**Figure 3:**
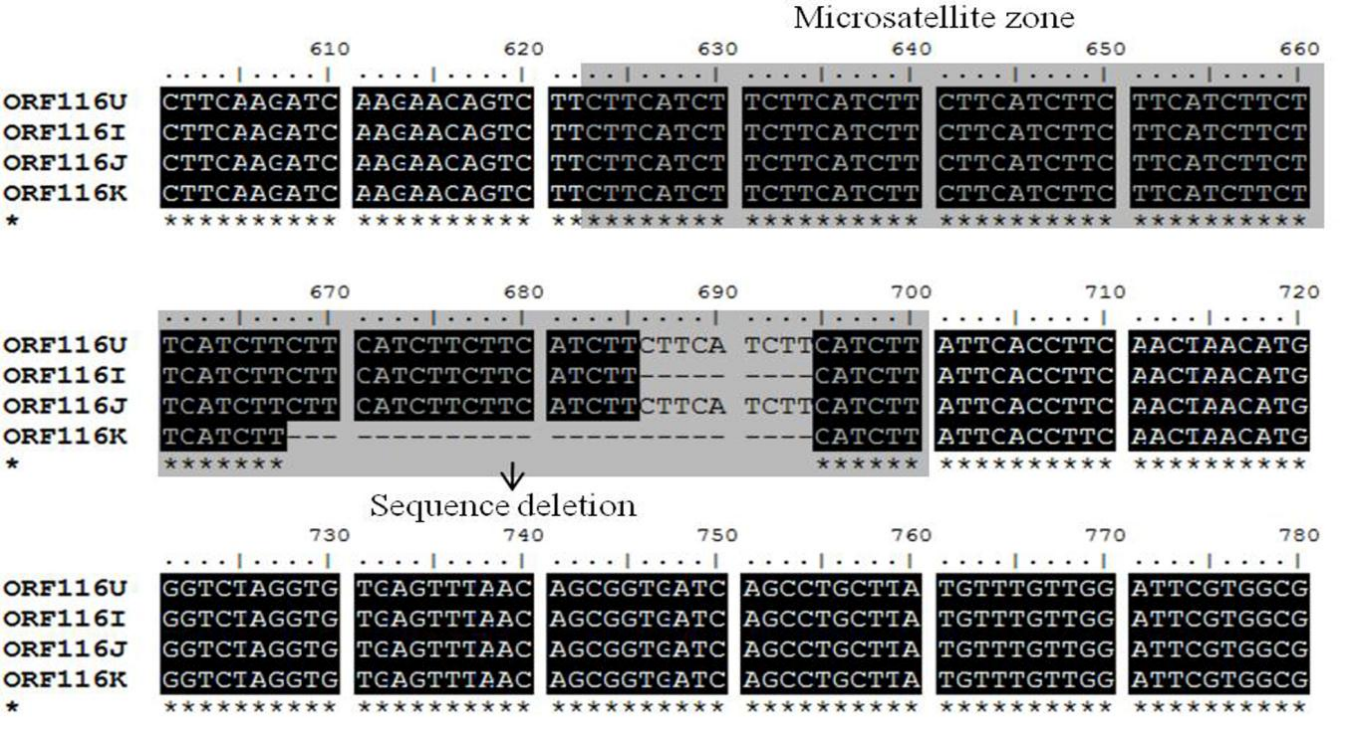
Nucleotide sequences of the glycoprotein gene (open reading frame [ORF]116) of Korean CyHV-3 (KHV-K) and those of the USA (KHV-U), Israel (KHV-I), and Japan (KHV-J) CyHV-3 were aligned using BioEdit software. Several sequence deletions were observed in the microsatellite zone in KHV-K.

Three selected regions of envelope glycoprotein genes from the Korean CyHV-3 were amplified and sequenced. These genes, encoded by ORF25, ORF65 and ORF116, are considered to encode the major envelope glycoproteins of this virus [7]. From the sequence analysis results, the ORF25, ORF65, and ORF116 regions of CyHV-3 from Korea, the USA, Israel, and Japan showed some sequence variations including several additions and deletions (Figure 1, Figure 2, Figure 3).

In the ORF65 region from Korean CyHV-3, the sequence insertion consisted of three consecutive nucleic acids in a repeated region of trinucleotides characterized by a “CAC” pattern, and the sequence deletion consisted of three consecutive nucleic acids in a repeated region of trinucleotides characterized by a “ACC” pattern (microsatellite zone) (Figure 2). Additionally, the microsatellite zone, characterized by a “CTTCATCTT” pattern, presented in ORF116, and a sequence deletion consisting of 27 consecutive nucleic acids in this repeated region was observed in Korean CyHV-3 (Figure 3).

Polymorphisms were also observed amongst American, Israeli, and Japanese CyHV-3 in these microsatellite zones; therefore, the microsatellite polymorphism of CyHV-3 determined in this study may be suitable for many applications including isolates differentiation and phylogenic studies. Microsatellites have been used to characterize different isolates, particularly in herpes cytomegaloviruses [13,14] and more recently in herpes simplex viruses [15] and in Ostreid herpes virus 1 [16].

### 2.2. Phylogenetic Analysis of CyHV-3 Envelope Glycoprotein Genes

**Figure 4:**
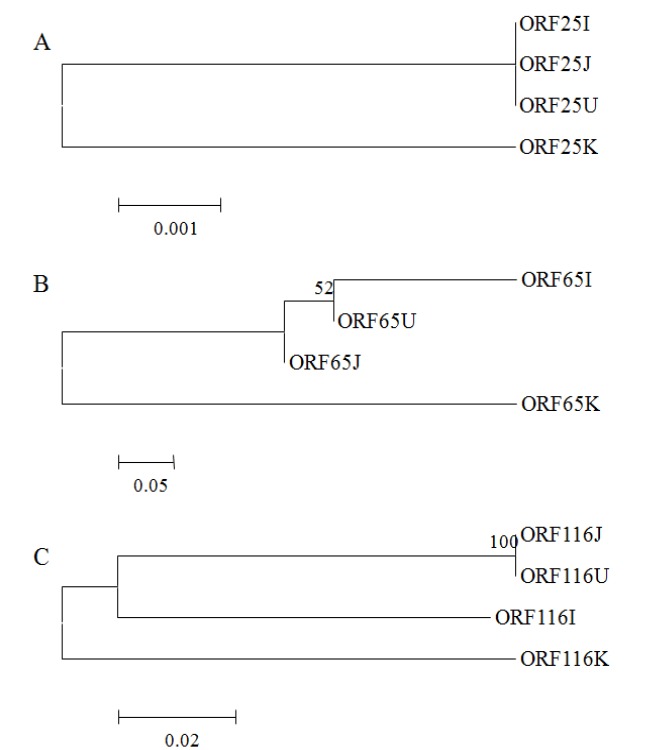
Phylogenetic trees were conducted on the sequences of the glycoprotein gene (open reading frame [ORF]25, ORF65 and ORF116)from Korea (KHV-K), the USA (KHV-U), Israel (KHV-I), and Japan (KHV-J) CyHV-3.

In phylogenetic trees (Figure 4A-C), sequences from the USA (KHV-U), Israel (KHV-I), and Japan (KHV-J) CyHV-3 were clustered into one group, but amplified sequences from the Korean CyHV-3 (KHV-K) was highly distinguished from this group.

In that previous report [17], a CyHV-3 isolate from Korea was compared with known CyHV-3 isolates and clustered into the same group based on sequence similarity (100%). A phylogenetic analysis based on the conserved region (ORF90) did not genetically distinguish the Korean isolate from the reference CyHV-3 isolates. However, envelope glycoprotein gene regions targeted in this study showed polymorphisms, indicating that the Korean CyHV-3 isolate was genetically different from those of the USA, Israel, and Japan isolates.

## 3. Experimental Section

### 3.1. Virus and Viral DNA

Viral DNA was extracted from the gill of koi [17] using the DNeasy Tissue Extraction kit (Qiagen, Valencia, CA, USA) according to the manufacturer’s instructions, and samples were stored at -80°C until use.

### 3.2. Amplification of Korean CyHV-3 Glycoprotein Genes

To acquire the full-length open reading frame (ORF) of potential envelope glycoprotein genes, three representative regions (ORF25, ORF65, and ORF116; 1,806 bp, 1,791 bp, and 855 bp, respectively) were chosen. Primers (Table 1) were designed from three full CyHV-3 genome sequences from the USA (KHV-U), Israel (KHV-I), and Japan (KHV-J), which were available in GenBank (DQ657948, DQ177346, and NC009127, respectively).

The polymerase chain reaction (PCR) reaction was performed in a final volume of 50 μl containing 100 ng total genomic DNA, 5 µl 10 × Taq-Buffer, 4 µl 10 mM dNTP’s, 1 U of platinum Taq DNA polymerase (Invitrogen, Carlsbad, CA, USA) and 2 pmol of each primer. After initial denaturation at 95 °C for 30 s, amplification was conducted for 30 cycles under the following conditions: 30 s at 95 °C, 1 min at the annealing temperature, and 1.5 min at 72 °C. Cycling was followed by a final extension for 7 min at 72 °C. Annealing temperatures of 62 °C, 63 °C, and 48 °C were used to amplify ORF25, ORF65, and ORF116, respectively. The PCR products were purified using a QIAquick PCR purification kit (Qiagen), according to the manufacturer’s instructions. Purified PCR products were ligated into the pTOP TA V2 plasmid vector using a TOP Cloner PCR Cloning kit (Enzynomics, Seoul, Korea), and this plasmid was transformed into competent DH5α (Enzynomics). Recombinant plasmids were extracted via the alkaline lysis procedure [18] and sequencing was carried out by the Macrogen Genomic Division, Seoul, Korea using ABI PRISM Big Dye TM Terminator Cycle Sequencing technology (Applied BioSystems, Foster City, CA, USA). Electrophoresis of sequencing reactions was completed using an automated ABI PRISM 3730XL DNA Sequencing System (Applied BioSystems). The sequences were subsequently analyzed with the AlignX tool in the Vector NTI program (Invitrogen). BLAST searches were carried out using both the blastn and blastx algorithms [19] against the National Center for Biotechnology Information.

### 3.3. Analysis of Korean CyHV-3 Glycoprotein Genes

Alignment of the glycoprotein gene nucleotide sequences of the Korean CyHV-3 (KHV-K) with those of the USA (KHV-U), Israel (KHV-I), and Japan (KHV-J) CyHV-3 were generated using BioEdit software version [20].

Also, the phylogenetic analysis was conducted using Bioedit software and Molecular Evolutionary Genetics Analysis (MEGA) 5 software, with bootstrap values calculated from 1000 replicates. The neighbor-joining method was used to construct the phylogenetic tree.

**Table 1:** Primers used in this study.

Primer name	Sequence (5’ → 3’ )	Purpose
ORF25		
25F1	ATGACGGGTTGTGGGGTTTGG	Full length CDS
25R1	TTAGGGCCTCCGGGAAACCTG	
25(m)F1	GCGTCTCGGGAGATACTTTG	Internal region
25(m)R1	GGGCACTCCATCTCAAAGAC	
ORF65		
65F1	ATGGTCTCGCCGCTCGTCGTC	Full length CDS
65R1	CTACTTGATGGTCGCGGCGGC	
ORF116		
116F1	ATGAGACTTTTTCTCCTCGTC	Full length CDS
116R1	TCAAACTTTTGGTGATGAAAA	

### 3.4. Nucleotide Sequence Accession Numbers

Newly amplified sequences of glycoprotein genes of Korean CyHV-3 were deposited in the GenBank nucleotide sequence database with the following accession numbers: JQ308816 for ORF25, JQ308817 for ORF65, and JQ308819 for ORF116.

## 4. Conclusions

Glycoprotein gene regions targeted in this study showed polymorphisms, indicating that the Korean CyHV-3 isolate was genetically different from those of the USA, Israel, and Japan isolates. Amplified sequences of the Korean CyHV-3 isolate contained several sequence insertions or deletions compared to those of reference CyHV-3. The ORF116 region presented as a sequence insertion (12 bp) in a microsatellite area of CyHV-3 from Korea. In addition, the ORF65 region had an insertion (3 bp) and a deletion (3 bp) in two microsatellite areas. As the data in microsatellite zones, amplified glycoprotein gene sequence of the Korean CyHV-3 (KHV-K) was distinguished from those of the USA (KHV-U), Israel (KHV-I), and Japan (KHV-J) CyHV-3 in the phylogenetic tree.

In this study, we successfully identified the putative envelope glycoprotein genes of CyHV-3 and analyzed sequences showed polymorphisms in microsatellite zones. These data could provide the genetic background for epidemiology studies. 
